# Reply to: Vitamin C as a promoter of γδ T cells

**DOI:** 10.1038/s41423-020-00622-3

**Published:** 2021-01-12

**Authors:** Léonce Kouakanou, Christian Peters, Dieter Kabelitz

**Affiliations:** Institute of Immunology, Christian-Albrechts University of Kiel and University Hospital Schleswig, Holstein Campus Kiel, 24105 Kiel, Germany

**Keywords:** Lymphocytes, Biomarkers

Last year, we published a paper in this journal in which we described the effects of vitamin C and its phospho-modified version (phospho-vitamin C, pVC) on the in vitro activation, proliferation, and effector function of human γδ T cells.^1^ We were delighted to see that our study was recently highlighted in a Comment in this journal by Meraviglia and Dieli,^2^ underscoring the potential interest in using vitamin C to promote γδ T cell activity for use in cancer immunotherapy. Our study was a joint publication with our Chinese collaborators, headed by Prof. Zhinan Yin, with whom we share an interest in exploring the beneficial effects of vitamin C on boosting the cellular immune response. In fact, Yin and colleagues initiated clinical studies on the adoptive transfer of allogeneic γδ T cells expanded in the presence of vitamin C into cancer patients. A report of a clinical phase I study demonstrating the safety of allogeneic γδ T cell transfer has recently been published in this journal, another collaborative effort of our two groups.^3^

In their comment, Meraviglia and Dieli highlight the most important results of our study without even mentioning our group. The results related to the rescuing effect of vitamin C on phosphoantigen-restimulated γδ T cells that were discussed at length in the Comment and summarized in their Fig. 1 were entirely performed by the first author of our paper as part of a Ph.D. thesis in our group. It would have been appropriate to mention in the legend of their Fig. 1 that the figure is based on results reported in our paper.^1^

Meraviglia and Dieli also comment on another recent paper by “the same authors”, in which we showed that vitamin C induced hypomethylation of the *FOXP3* locus in human γδ T cells activated in the presence of TGF-β.^4^ In this context, the term “same authors” refers to their previous wording of “Yin and colleagues” throughout the Comment. Apparently, it has escaped the attention of Meraviglia and Dieli that Prof. Yin and our Chinese collaborators are not even coauthors on this paper.^4^

It is important for journals to highlight outstanding papers with Comments that summarize broader perspectives. However, authors of such Comments need to make sure that adequate credit is given to the original authors.
